# Epigallocatechin-3-gallate binds tandem RNA recognition motifs of TDP-43 and inhibits its aggregation

**DOI:** 10.1038/s41598-025-02035-6

**Published:** 2025-05-23

**Authors:** Maria Agnese Morando, Vito D’Alessandro, Angelo Spinello, Martina Sollazzo, Elisa Monaca, Raffaele Sabbatella, Maria Concetta Volpe, Francesca Gervaso, Alessandro Polini, Sarah Mizielinska, Caterina Alfano

**Affiliations:** 1 Structural Biology and Biophysics Unit, Fondazione Ri.MED, 90133 Palermo, Italy; 2 Department of Mathematics and Physics “E. De Giorgi”, University of Salento, 73100 Lecce, Italy; 3 Department of Biological, Chemical and Pharmaceutical Sciences and Technologies (STEBICEF), University of Palermo, 90028 Palermo, Italy; 4 Fondazione Ri.MED, 90133 Palermo, Italy; 5 CNR Nanotec-Institute of Nanotechnology, Campus Ecotekne, 73100 Lecce, Italy; 6 UK Dementia Research Institute at King’s College London, London, UK; 7 Department of Basic and Clinical Neuroscience, Institute of Psychiatry, Psychology and Neuroscience, King’s College London, London, UK

**Keywords:** EGCG stability, TDP-43, ALS, Protein aggregation, RNA-binding proteins, Protein-ligand interaction, Biophysics, Drug discovery, Structural biology

## Abstract

Transactive response DNA-binding Protein 43 (TDP-43) aggregation is a key pathological feature in Amyotrophic Lateral Sclerosis and related neurodegenerative diseases. This study investigates the inhibitory effects of Epigallocatechin-3-gallate (EGCG), a polyphenol found in green tea, on TDP-43 aggregation. Using a combination of fluorescence assays, NMR spectroscopy, and computational modeling, we demonstrate that Epigallocatechin-3-gallate significantly delays the nucleation phase of TDP-43 aggregation process, thus inhibiting the formation of TDP-43 aggregates *in vitro.* Additionally, we proved a direct interaction of the compound with the RNA recognition motifs of TDP-43 and modeled the mechanism of interaction. Our findings reveal that EGCG stabilizes the RRM domains, counteracting aggregation by interfering with the early stages of the amyloidogenic pathway. Furthermore, EGCG’s stability under experimental conditions was ensured using reducing agents, highlighting the importance of maintaining its reduced form for reproducible results. These insights underscore the therapeutic potential of EGCG in TDP-43 proteinopathies and provide a foundation for developing targeted treatments for ALS and related disorders.

**Supplementary Information:**

The online version contains supplementary material available at 10.1038/s41598-025-02035-6.

## Introduction

Amyotrophic Lateral Sclerosis (ALS) is a debilitating and fatal neurodegenerative disease characterized by progressive motor neuron degeneration.^1,2^ A pathological hallmark of ALS is the formation of cytosolic aggregates composed of poly-ubiquitinated and hyper-phosphorylated Transactive response DNA-binding Protein 43 (TDP-43) within neurons and glial cells.^3–5^ The significance of TDP-43 in ALS pathogenesis is further underscored by the identification of more than 50 mutations in the TDP-43 gene (TARDBP) associated with ALS, highlighting the central role of this protein in the disease.^6–12^ Notably, TDP-43 mutations are also implicated in frontotemporal lobar dementia (FTD), a neurodegenerative condition that shares clinical and pathological features with ALS. Approximately 45% of FTD cases also exhibit TDP-43 pathology, suggesting a common molecular underpinning between these two diseases.^3,5,13^

TDP-43 is a nuclear factor that plays a crucial role in gene expression, particularly in transcriptional regulation and alternative splicing. Structurally, this protein is a nucleic acid-binding protein that comprises a well-folded N-terminal domain, two conserved RNA recognition motifs (RRM1 and RRM2), and a prion-like C-terminal domain, with the latter harboring the majority of disease-associated mutations.^14–16^ In its normal physiological state, TDP-43 primarily resides in the nucleus, where it is involved in RNA metabolism and processing by binding to UG-rich RNA and single-stranded TG-rich DNA sequences through its RRM domains.^17^ However, pathologic TDP-43 is mislocalized in the cytoplasm, where it forms pathological aggregates in over 97% of cases.^18,19^ These aberrant cytoplasmic inclusions often colocalize with stress granules, which are transient membrane-less organelles composed of protein-RNA complexes formed in response to cellular stress.^20^

Multiple studies have identified the RRM domains of TDP-43 as critical players in aggregation and pathology, and amyloidogenic sequence elements are found in these domains.^21–28^ Moreover, it has been shown that binding with RNA plays a pivotal role in modulating the aggregation of TDP-43 RRMs, acting as either a facilitator or inhibitor of the process.^29–31^ For instance, tight binding with UG-rich RNA aptamers prevents fibrillation of TDP-43 constructs containing the N-terminal domain and both RRMs, whereas weak binding with non-UG-rich aptamers can accelerate its aggregation.^29^ The role of the RRMs in TDP-43 aggregation is further emphasized by studies showing that mutations disrupting RNA binding significantly enhance TDP-43 aggregation.^32^

All these findings suggest that stabilizing the RRM domains of TDP-43 is critical for maintaining the protein solubility and preventing its pathological aggregation.

In this study, we explored the potential of Epigallocatechin-3-gallate (EGCG), a polyphenol that constitutes more than 50% of the catechins found in green tea, to stabilize the RRM domains of TDP-43 and inhibit its aggregation. Several studies have reported that EGCG has neuroprotective effects for several neurodegenerative diseases,^33–36^ and potently inhibits the formation of huntingtin, abeta, and alpha-synuclein aggregates by redirecting these proteins into non-toxic, unstructured, off-pathway oligomers.^37–40^

In the context of TDP-43 pathological aggregation, previous studies indicate that EGCG may preserve the native conformation of the protein full-length and its C-terminal domain, and prevents TDP-43 degradation and toxic aggregation. Moreover, EGCG-induced TDP-43 aggregates have been reported to be non-toxic.^41,42^ However, the specific effects of EGCG on the RRM domains of TDP-43, which are central to its aggregation and function, remain largely unexplored.

Here, we investigate the inhibitory effects of EGCG on TDP-43 aggregation, focusing on its effect on the RRM domains. Using a combination of fluorescence assays, NMR spectroscopy, and computational modeling, we demonstrate that EGCG significantly delays the nucleation phase of TDP-43 aggregation and reduces the formation of amyloid-like aggregates. Our findings reveal that EGCG directly interacts with the RRM domains, stabilizing them in a non-aggregating state. Additionally, we highlight the importance of maintaining EGCG in its reduced and stable form during experiments, as its degradation can confound results. These insights extend our understanding of the mechanism of action of EGCG and its therapeutic potential in TDP-43 proteinopathies.

By elucidating the molecular basis of EGCG’s interaction with TDP-43, we aim to contribute to the development of targeted therapies for ALS and related disorders. The findings presented in this study not only reinforce the therapeutic potential of EGCG but also emphasize the importance of understanding the structural and functional dynamics of TDP-43. The interplay between RNA binding, protein aggregation, and cellular stress responses remains a critical area of investigation, with implications for a wide range of neurodegenerative conditions. Future research should focus on integrating biophysical insights with translational studies to develop effective interventions for TDP-43-associated diseases.

## Results

### EGCG inhibits *in vitro* TDP-43 RRMs aggregation

We first investigated whether EGCG has a modulatory effect on the formation of TDP-43 aggregates *in vitro*. To this purpose, a recombinant TDP-43 construct composed of both RRM1 and 2 (TDP-43 RRMs) was used in a Thioflavin T (ThT)-binding assay, ThT being widely used as a fluorescent probe for detecting amyloid-like aggregates.^43^ The aggregation assay was performed in parallel with increasing concentrations of EGCG ranging from 0 to 30 µM, corresponding to protein: EGCG ratios 1:0, 1:0.5, 1:1, and 1:2. Although literature reports that oxidized EGCG may enhance its anti-aggregation efficacy,^44–46^ in our aggregation assay EGCG was kept in its reduced and stable form by supplementing the sample buffer with 1,4-Dithiothreitol (DTT). This decision was driven by the known instability of EGCG,^47,48^ which we investigated further in our experimental conditions by a 1DH NMR time course stability analysis. Specifically, we acquired NMR spectra over time on EGCG samples with and without the addition of a reducing agent, which revealed that EGCG rapidly degrades in the absence of a reducing agent while maintaining its reduced and stable form when in the presence of a reducing agent (Suppl. Fig. 1).

**Fig. 1:**
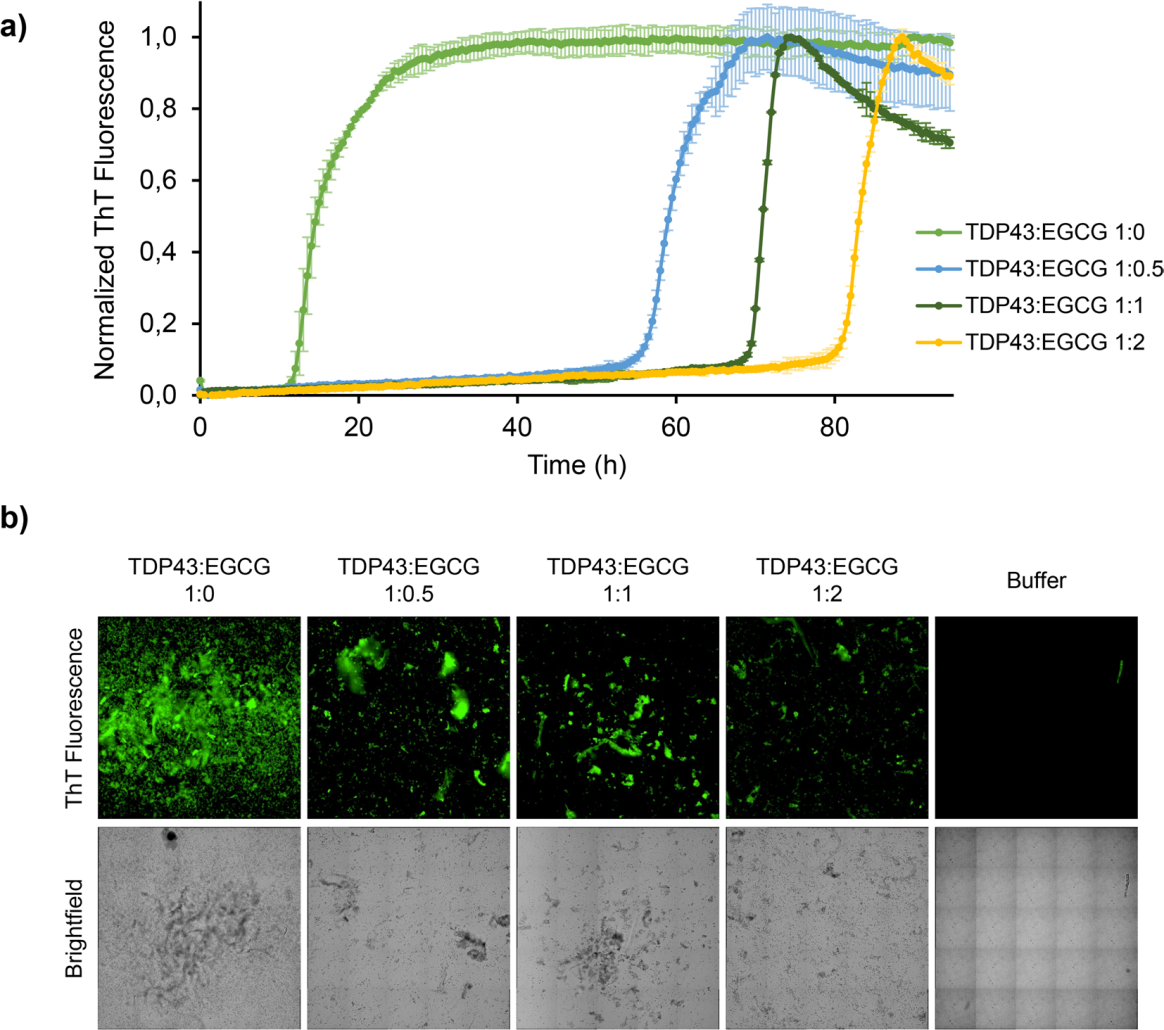
Effect of EGCG on TDP-43 RRMs aggregation. (**a**) Time evolution of the ThT fluorescence intensity for 15 µM TDP-43 RRMs samples incubated at 37 °C for ~ 90 h with increasing concentration of EGCG ranging from 0 to 30 µM, corresponding to protein:EGCG ratio 1:0, 1:0.5, 1:1, and 1:2. Each curve represents the mean of triplicate measurements normalized to the maximum reached value. Error bars in each curve represent the standard deviation of replicates. (**b**) Inspection of the ThT aggregation kinetics endpoints by fluorescent microscopy (top) and brightfield microscopy (bottom).

The ThT assay results provided compelling evidence that EGCG exerts a significant inhibitory effect on the aggregation of TDP43 RRMs *in vitro*. Both the control and EGCG-treated samples exhibited a characteristic sigmoidal ThT fluorescence curve, indicative of nucleation-mediated aggregation, which is typical for amyloid-forming proteins (Fig. 1a). However, marked differences were observed between the two conditions. Without EGCG, the lag phase of aggregation was approximately 10 h. In contrast, in the presence of EGCG, this phase was drastically extended as a function of the EGCG concentration, clearly indicating that EGCG interferes with a very early step in the amyloid formation pathway and suppresses the assembly of on-pathway amyloidogenic oligomers and protofibrils.^49^ Additionally, we inspected the endpoints of the ThT aggregation kinetics using both brightfield and fluorescent microscopy. Large objects several micrometers in size were visible in all samples but reduced in number in the presence of EGCG. Moreover, ThT fluorescence intensity was substantially lower in the EGCG-treated samples, indicating that EGCG reduces the amyloid-like aggregates content (Fig. 1b). These results align with previous studies that have shown the ability of EGCG to modulate protein aggregation.^35,40–42^

Taken together, our findings prove an inhibitory effect of EGCG on TDP-43 RRMs aggregation.^41,42^ Furthermore, our results highlighted a critical aspect often overlooked in EGCG research: the compound’s stability under experimental conditions. Our 1D ^1^H NMR analysis revealed that EGCG begins to degrade within 1 h in the absence of a reducing agent in the sample buffer. This evidence underscores the importance of maintaining EGCG in its reduced form to affect TDP-43 aggregation, as well as the importance of verifying the structure and stability of any component to assure the rigor of the investigation.

### EGCG exhibits a direct interaction with TDP-43 RRMs

To investigate whether the inhibitory effect of EGCG on TDP-43 RRMs aggregation is mediated by a direct interaction between the two molecules, we employed Saturation Transfer Difference (STD) and Transferred Nuclear Overhauser Enhancement (trNOE) NMR experiments. These well-established and versatile techniques are ideal for detecting weak protein-ligand interactions using small quantities of non-labeled proteins.^50^

**Fig. 2:**
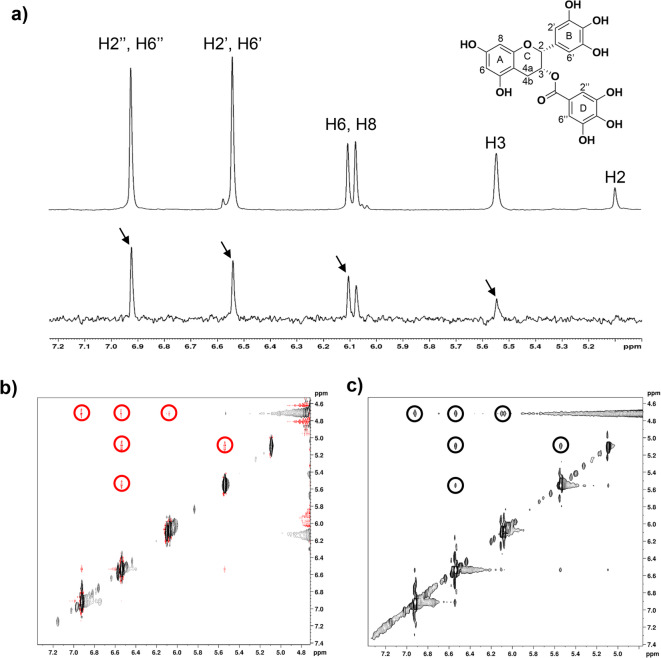
Interaction between EGCG and TDP-43 RRMs proved by NMR spectroscopy. (**a**) Comparison between the 1D ^1^H NMR reference spectrum of free EGCG (top) and STD spectrum of EGCG in the presence of TDP-43 RRMs (bottom). The structure of EGCG is reported together with the assignment of the proton signals. The presence of EGCG signals in the STD spectrum (black arrows) indicates a binding between the ligand and the protein. (**b**) 2D NOESY spectrum of free EGCG. Cross (highlighted in red circles) and diagonal peaks have opposite signs as expected for a small molecule in a free state. (**c**) trNOE spectrum of EGCG in the presence of TDP-43 RRMs. Cross (highlighted in black circles) and diagonal peaks have the same sign as expected for a ligand bound to a large molecule.

The STD-NMR spectrum revealed signals from the ligand protons, indicating a direct binding event between EGCG and TDP-43 RRMs (Fig. 2a).^51^ Further validation was provided by acquiring a 2D NOE Spectroscopy (NOESY) NMR experiment on free EGCG, and a trNOE NMR experiment on EGCG in the presence of TDP-43 RRMs. The 2D NOESY spectrum of free EGCG displayed the expected NOE pattern, with cross and diagonal peaks of opposite signs, characteristic of a small molecule in its free state. Conversely, in the trNOE spectrum of EGCG in the presence of TDP-43 RRMs, the cross and diagonal peaks showed the same sign, consistent with the behavior expected for a ligand bound to a large molecule (Fig. 2b,c).^52,53^

Taken together, our results indicate a specific, although weak, interaction between EGCG and TDP-43 RRMs. This interaction does not induce significant conformational changes in EGCG compared to its unbound form, and the primary sites of interaction on the ligand appear to be the aromatic rings. More investigation is needed to assess the contribution of the hydroxyl groups linked to the aromatic rings.

### 3D structural model of RNA-free TDP-43 RRMs

To identify the EGCG binding site on TDP-43 RRMs, we modelled the protein structure using the deep learning-based prediction tool RoseTTAFold.^54^ This was necessary because the only available experimental structure of TDP-43 RRMs is in complex with UG-rich RNA (PDB 4bs2).^55^ In this structure, the relative orientation of the two RRM domains is stabilized by interactions with the RNA, resulting in a well-defined conformation. However, this RNA-bound conformation differs from the structure of the RNA-free protein in which the highly flexible loop between the two RRMs is thought to confer adaptability to different nucleic acid partners by allowing different orientations of the RRM domains.^56,57^

**Fig. 3:**
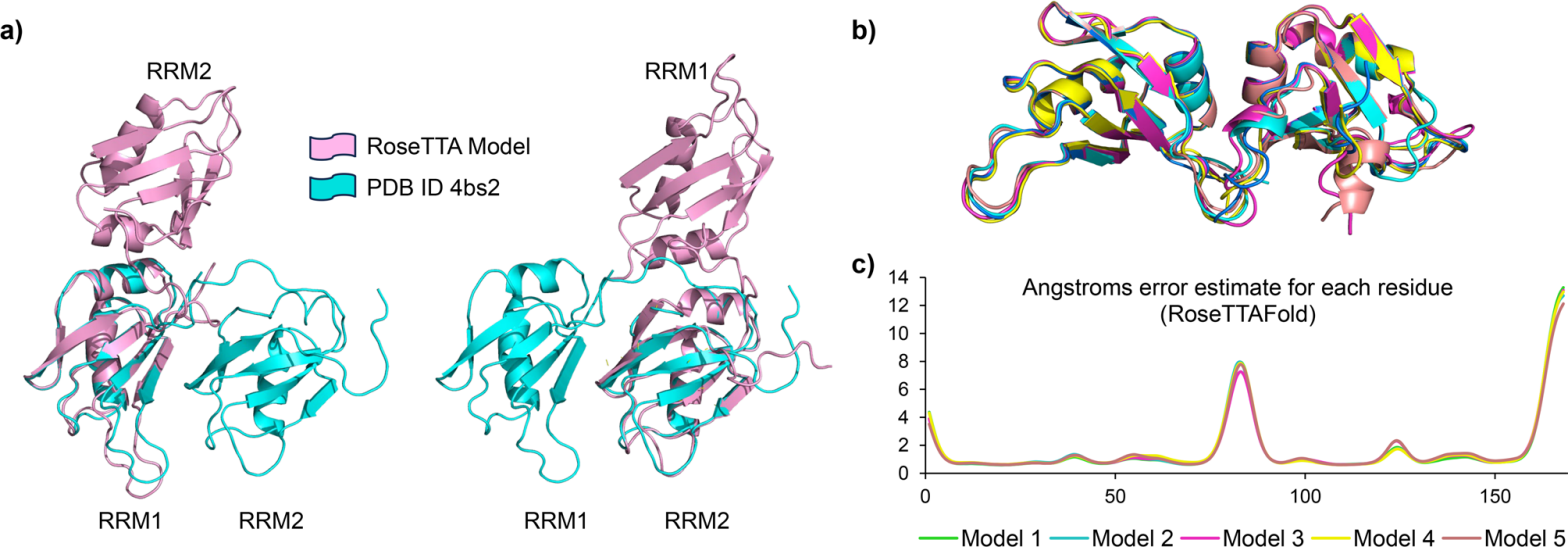
3D structural model of free TDP-43 RRMs. (**a**) Overlap between the RoseTTAFold model 1 of RNA-free TDP-43 RRMs (pink) and experimental RNA-bound structure (cyan, PDB 4bs2). The alignment was performed by overlapping, in turn, RRM1 (left) and RRM2 (right). The RoseTTAFold model and the experimental RNA-bound structure share the overall structure of the two RRMs. Nonetheless, these display a different mutual orientation. (**b**) Cartoon representation of the 5 best-scored models generated by RoseTTAFold, in overlap. (**c**) Angstrom error estimates versus the position of each residue in the sequence.

The RoseTTAFold model obtained for RNA-free TDP-43 RRMs maintains the overall structure of the two RRMs in the experimental RNA-bound structure, but differs for the mutual orientation of the two RRMs, with the RoseTTAFold model displaying a more open conformation (Fig. 3a). The five best-scored models showed high confidence values (0.81) and consistent secondary structure elements, as well as a similar mutual orientation of the two RRMs. Higher error values were obtained for the 14-amino acid linker between the two RRM domains, and in the C-terminal tail (Fig. 3b,c). Consistently, residues in these two regions showed lower I/I_0_ values in the ^1^H–^15^N heteronuclear NOE NMR relaxation experiment, indicating that they experience movements in the nanosecond to picosecond timescale (Suppl. Fig. 2.)^58^

**Fig. 4:**
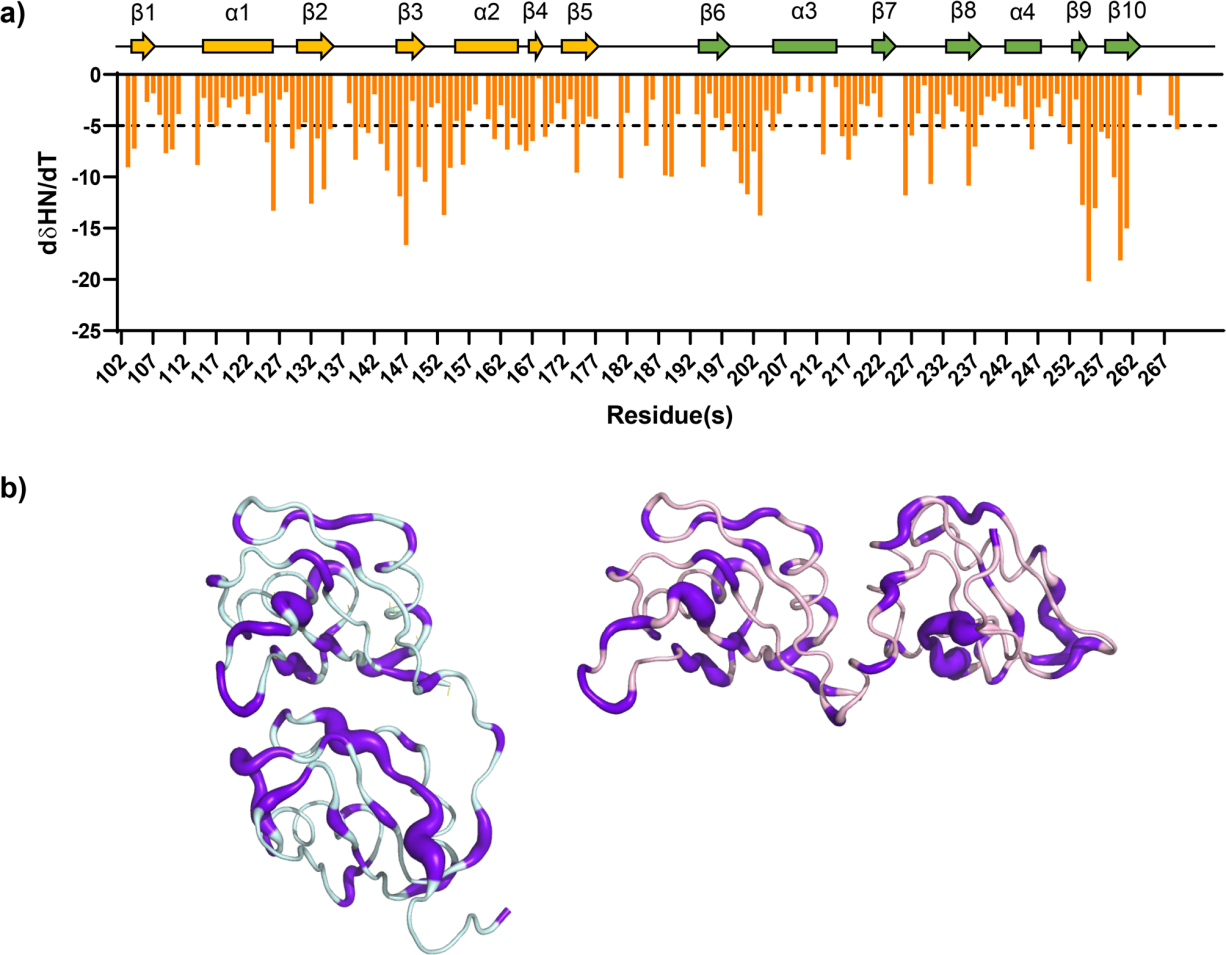
Amide chemical shift temperature coefficients analysis. (**a**) Temperature coefficient values versus the position of each residue in the sequence. Values lower than − 5 ppb/K (threshold in dotted line), are typically associated with solvent-exposed amide protons forming intermolecular hydrogen bonds with water. (**b**) Worm representation of RNA-bound structure (cyan) and RoseTTAFold model 1 of RNA-free TDP-43 RRMs (pink). Residues experiencing temperature coefficient values lower than − 5 ppb/K are indicated in purple in both structures. The thickness of the purple traits linearly correlates to the dδHN/dT value.

To validate the RoseTTAFold model of RNA-free TDP-43 RRMs, we measured the amide chemical shift temperature coefficients by recording a series of ^1^H–^15^N HSQC spectra at increasing temperatures (Fig. 4a). This approach evaluates the solvent accessibility of amide protons, and thus their propensity to form inter- and intramolecular hydrogen bonds.^59^ Temperature coefficient values lower than − 5 ppb/K, typically associated with solvent-exposed amide protons forming intermolecular hydrogen bonds with water, were mapped onto both the RoseTTAFold model and the experimental RNA-bound structure for comparison (Fig. 4b). RoseTTAFold model 1 was arbitrarily selected for this comparison. We found that the RoseTTAFold model is consistent with the experimental NMR data, as residues with temperature coefficient values lower than − 5 ppb/K were exposed to the solvent. In contrast, these same residues were located at the interface between the two RRMs when mapped onto the RNA-bound experimental structure. These findings suggest that the RNA-free TDP-43 RRMs adopts a more flexible and open conformation compared to its RNA-bound state, highlighting the dynamic nature of TDP-43 and its potential structural adaptability in the absence of RNA.

### Characterization of EGCG binding sites on TDP-43 RRMs

**Fig. 5:**
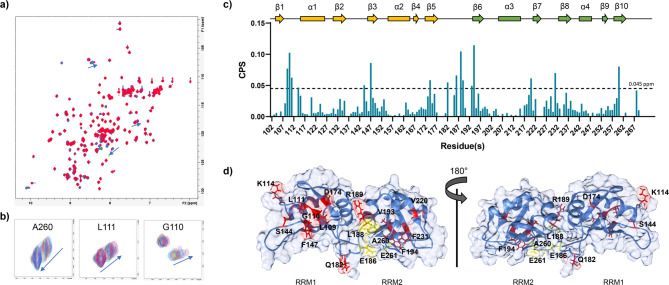
Chemical shift perturbation analysis. (**a**) Overlay of 2D ^1^H–^15^N-HSQC spectra of TDP-43 RRMs upon EGCG titration. (**b**) Highlight of representative ^1^H–^15^N-HSQC peaks exhibiting significant perturbation upon EGCG titration. (**c**) Chemical shift perturbation values for each amino acidic residue of TDP-43 RRMs. (**d**) Cartoon representation of the RoseTTAFold model 1 of TDP-43 RRMs. Highlighted in red those residues experiencing CSP > 0.045 ppm, while in yellow those residues for which the correspondent peak in the ^1^H–^15^N-HSQC seems to disappear upon EGCG titration.

To identify the EGCG binding site on TDP-43 RRMs, we performed Chemical Shift Perturbation (CSP) NMR analysis^60^. ^15^N-labeled TDP-43 RRMs was titrated with increasing concentrations of EGCG, and 2D ^1^H–^15^N HSQC spectra were recorded for each titration point to track resonance shifts (Fig. 5a). Several peaks shifted smoothly as a function of EGCG concentration, indicating a fast exchange regime between the free and bound states (Fig. 5b). The observed CSPs were quantified, and residues with significant shifts, indicative of potential binding events, were mapped onto the RoseTTAFold model of TDP-43 RRMs (Fig. 5c,d). Notably, the most substantial shifts were observed for residues localized in two defined regions, each localized on one of the two RRMs, suggesting a protein: ligand stoichiometry of 1:2. Additional CSPs were observed in the flexible 14-amino acid linker. However, these were likely due to conformational changes rather than direct ligand binding, as suggested by ^15^N backbone amide relaxation parameters measured after the CSP experiments (Suppl. Fig. 3).

**Fig. 6:**
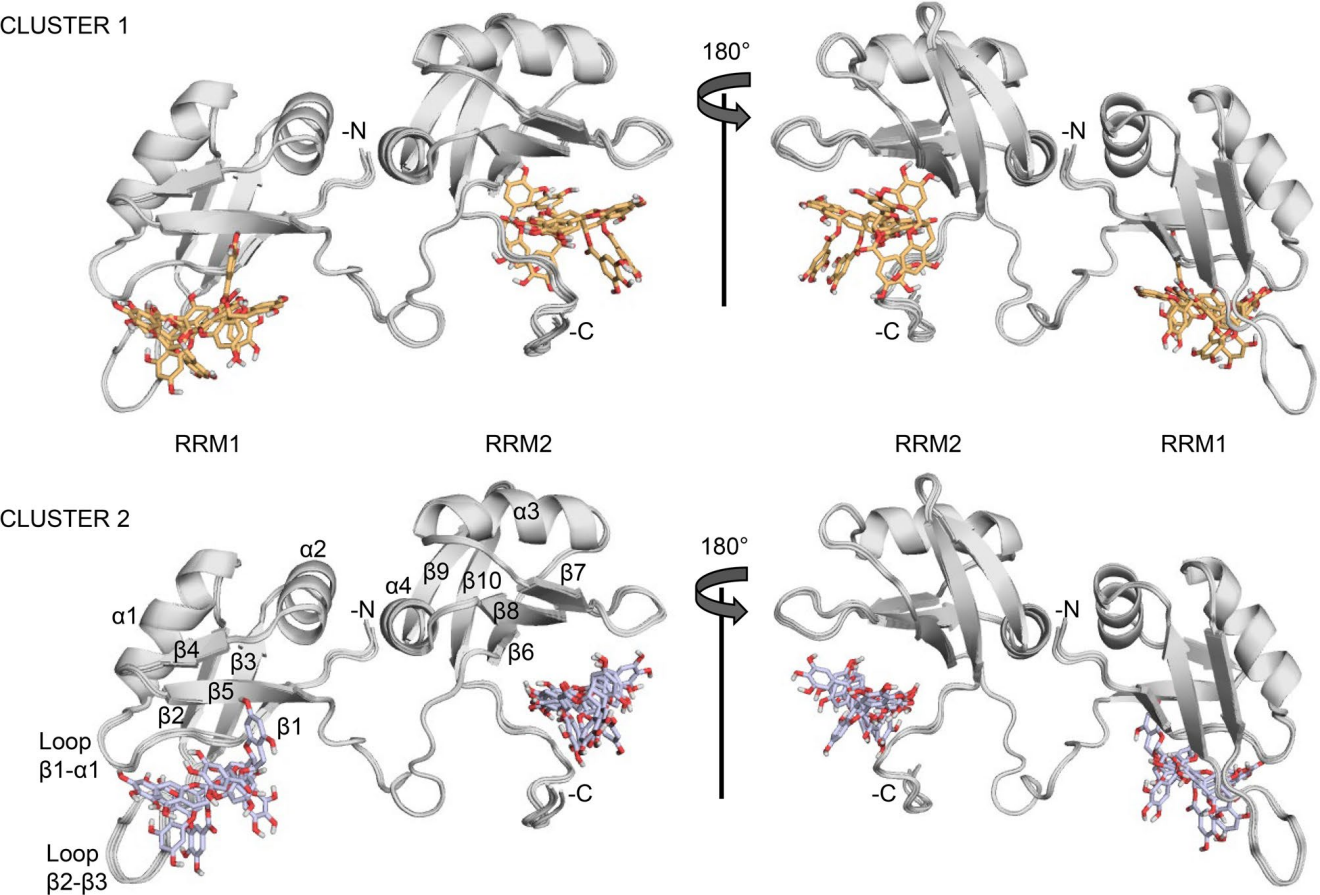
Docking models. Cartoon superimposition of 4 of the most representative models from both HADDOCK Cluster 1 (top) and 2 (bottom). The two clusters show similar EGCG binding sites on RRM1 and RRM2. However, no well-defined binding pockets for EGCG are observed, allowing the ligand to adopt slightly different binding poses across simulations.

CSP-guided docking calculations by the HADDOCK suite^60,61^ were then performed imposing a protein:ligand stoichiometry of 1:2 and binding restraints only for those residues laying in structured regions. The HADDOCK output generated 190 structures grouped into eight clusters, with Clusters 1 and 2 being the most populated and having similar HADDOCK scores: − 54.6 ± 1.8 and − 54.9 ± 2.0, respectively (Table 1). Both clusters displayed low restraint violation energies, indicating that the CSP-derived restraints were well accommodated. Superimposition of the best structures of each cluster revealed similar EGCG binding sites on RRM1 and RRM2, with recurrent interactions between the proteins and ligands (Table 2). However, no well-defined binding pockets for EGCG were observed, allowing the ligand to adopt slightly different binding poses across simulations (Fig. 6).

**Table 1:** HADDOCK outputs of the most populated clusters obtained by TDP-43 RRMs/EGCG docking.

	Cluster 1	Cluster 2
Haddock score	− 54.6 ± 1.8	− 54.9 ± 2.0
Cluster size	91	48
RMSD from overall lowest energy structure	0.4 ± 0.0	0.3 ± 0.1
Van der Waals energy	− 47.3 ± − 2.6	− 48.2 ± − 2.3
Electrostatic energy	− 29.1 ± − 18.9	− 19.4 ± − 24.5
Desolvation energy	− 6.0 ± − 3.4	− 5 ± − 3.2
Restraints violation energy	16.4 ± − 15.04	2.4 ± − 1.29
Z-score	− 1.0	− 1.1

**Table 2:** Recurrent interactions observed in HADDOCK Clusters 1 and 2.

	Cluster 1	Cluster 2
RRM1	Hydrogen bond G110 (HN) L111 (HN) W113 (NH) H143 (NH) S144 (C=O) K145 (C=O) G146 (C=O) R165 (NH2) R171 (NH2) D174 (C=O) π-cation R171	Hydrogen bond G110 (C=O) L111(C=O) (HN) H143 (NH) S144 (C=O) (OH) K145 (C=O) G146 (C=O) R171 (NH2) D174 (C=O) π-cation R171
RRM2	Hydrogen bond N259 (C=O) E261 (C=O) (HN) K263 (NH3) H264 (NH) π-cation K263 π–π F194 F231	Hydrogen bond K192 (NH3) E261 (C=O) (HN) K263 (HN) (NH3) H264 (NH) R268 (NH2)

**Fig. 7:**
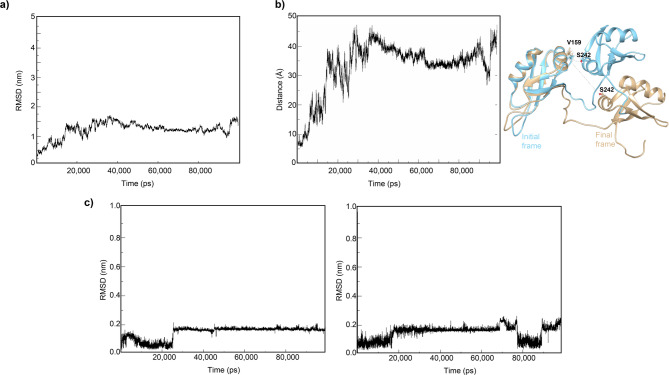
Parameters from molecular dynamics simulation on HADDOCK Cluster 1. (**a**) RMSD for the protein structure over 100ns of simulation from the starting HADDOCK model. (**b**) Distance between Cα of V159 and Cα of S242 measured along the simulation. The two RRM domains assume different orientations with respect to the initial frame, moving away from each other. This distance correlated with the RMSD fluctuations observed in (**a**). (**c**) RMSD along the 100ns simulation of EGCG molecule bound to RRM1 (left) and RRM2 (right).

To assess the stability of the docked structures, we conducted two separate all-atom molecular dynamics (MD) simulations using GROMACS,^62^ one for each top-scoring structure from Clusters 1 and 2. Both simulations confirmed the persistence of EGCG binding to the protein throughout the trajectory. The Root Mean Square Deviation (RMSD) analysis of the protein in complex with EGCG revealed fluctuations, primarily due to the dynamic nature of the 14-amino acid linker between the two RRMs, which influences their relative orientation. Fluctuations were also observed when looking at the RMSDs of the two EGCG molecules bound to the protein, as expected when a well-defined binding pocket is missing (Fig. 7 and Suppl. Fig. 4).

To pinpoint the protein residues most involved in binding, we analyzed the frequency of hydrogen bonds and the presence of π–π or cation–π interactions. The analysis revealed that EGCG engages more stably with RRM1 than RRM2. In RRM1, 10 hydrogen bonds were formed during the simulation, with residues L111, S144, D174, and D185 being the most involved, showing occupancies of 61.57%, 13.21%, 42.38%, and 78.59%, respectively. Additionally, F147 formed a transient π–π interaction with EGCG’s aromatic rings A and D. In RRM2, the total number of hydrogen bonds fluctuated around 5 throughout the simulation, with residues D247 and K263 having the highest occupancies at 7.21% and 21.98%, respectively. Aromatic side chains of residues F194, F231, and H264 were instead involved in π-π interactions with the EGCG aromatic rings A and B.

These findings suggest that, while EGCG binds to both RRM1 and RRM2 of TDP-43, the interaction is more stable with RRM1. Moreover, the structural stability of the protein-ligand complex and the overall binding dynamics are influenced by the flexibility of the 14-amino acid linker, which plays a significant role in modulating the relative orientation of the two RRMs.

## Discussion

The present study explores the inhibitory effect of EGCG on the aggregation of TDP-43, a protein critically implicated in the pathogenesis of ALS and other neurodegenerative diseases. Despite extensive research, the precise mechanisms driving TDP-43 aggregation and its toxic effects remain incompletely understood. However, the involvement of specific domains of TDP-43, such as its RRM domains, has been well documented.^21–27,29,32^ These RRMs are critical for the protein’s interaction with RNA and are also implicated in its aggregation propensity. Consequently, understanding how small molecules like EGCG can modulate the behavior of these domains offers valuable insights into potential therapeutic approaches for ALS and other TDP-43-related pathological conditions.

On the other hand, EGCG has attracted considerable attention as a phytochemical showing promise in modulating protein aggregation toxicity.^33–36,63^ The anti-aggregation properties of EGCG are thought to stem from its ability to stabilize proteins in their native or soluble oligomeric states, thereby preventing the formation of toxic aggregates. Moreover, EGCG has been reported to remodel existing aggregates into non-toxic forms, highlighting its potential not only as a preventive but also as a therapeutic agent.^37–40,44^

In the context of TDP-43 self-assembly, previous research has shown that EGCG can modulate the aggregation of both full-length and C-terminal domain of TDP-43.^41,42^ However, the specific effects of EGCG on the RRMs of TDP-43, which are involved in the protein’s aggregation and function, have not been thoroughly explored until now. Our study provides new insights into how EGCG interacts with these domains and the implications for TDP-43 aggregation. More specifically, our study revealed that EGCG exerts a significant inhibitory effect on the aggregation of TDP-43 RRMs *in vitro*. Using a ThT-binding assay, we demonstrated that EGCG considerably delays the nucleation phase of aggregation and reduces the overall formation of ThT-positive aggregates. The extension of the lag phase and the reduction of the maximum fluorescence intensity observed in our ThT assay suggest that EGCG interferes with the early stages of TDP-43 aggregation, potentially by binding to the protein and stabilizing it in a non-amyloidogenic form. Accordingly, our study revealed a direct albeit weak interaction between EGCG and TDP-43 RRMs.

The 3D structural analysis of the RNA-free TDP-43 RRMs, predicted using RoseTTAFold, provided further context for the observed interactions. The model revealed that TDP-43 RRMs adopts a more open and flexible conformation in the absence of RNA than in the RNA-bound state. This flexibility is particularly pronounced in the 14-amino acid linker between RRM1 and RRM2, which is likely to influence the overall dynamics of the protein and its interaction with ligands like EGCG. Our docking studies and molecular dynamics simulations supported this notion, showing that EGCG can bind to both RRM1 and RRM2, with a preference for RRM1. This suggests that RRM1 may play a more crucial role in the aggregation process of TDP-43, and that targeting this domain with small molecules like EGCG could be particularly effective in preventing TDP-43 aggregation. Given that RRM1 plays a key role in RNA recognition, this raises the question of whether EGCG competes with RNA for binding. Our findings suggest that while some overlap exists between EGCG binding sites and RNA-binding regions, direct competition is highly unlikely. The dynamic nature of the TDP-43/EGCG interaction, combined with the flexible conformation of the 14-amino acid linker between RRM1 and RRM2, allows for multiple binding poses without necessarily displacing RNA. This observation aligns with previous studies showing that EGCG can engage structurally distinct regions of proteins without fully occluding their functional sites.^64^ The dynamic nature of the linker thus emerged as a critical factor influencing the binding of EGCG and the structural stability of the protein-ligand complex. The linker’s flexibility allows the two RRMs to adopt various relative orientations, which in turn affects how EGCG interacts with the protein. This observation is important because it highlights the potential for allosteric effects in the regulation of TDP-43 aggregation. By modulating the conformation of the linker, it may be possible to influence the overall aggregation behavior of TDP-43, providing a novel therapeutic strategy.

Our findings reinforce the potential of EGCG as a therapeutic agent in neurodegenerative diseases. By demonstrating that EGCG can directly interact with and inhibit the aggregation of TDP-43 RRMs, we provide a mechanistic basis for its neuroprotective effects observed in other models. This adds up to the growing body of evidence supporting the use of EGCG and other polyphenolic compounds in neurodegenerative disease therapy.

A key question in this study concerns how EGCG, a single small molecule, can engage with structurally distinct regions of TDP-43, namely the structured RRMs and the intrinsically disordered C-terminal domain. EGCG is known for its promiscuous binding properties, primarily mediated by π–π stacking and hydrogen bonding with aromatic residues, which can easily be engaged in both structured RRMs and the disordered C-terminal domain despite their structural differences. This mode of binding is not uncommon for amyloid-modulating small molecules. EGCG has been shown to bind proteins differently depending on their conformational state: in structured proteins, it preferentially interacts with dynamic, exposed loops, while in intrinsically disordered proteins, it binds more diffusely along the sequence^64^. This dual interaction model provides a plausible explanation for EGCG’s ability to engage multiple regions of TDP-43.

Our study highlights the importance of targeting specific domains of TDP-43 in therapeutic development. The differential binding of EGCG to RRM1 and RRM2 suggests that these domains may contribute to TDP-43 aggregation in distinct ways, confirming previous evidence.^27^ Therefore, therapies that selectively target RRM1 could potentially be more effective in preventing or reversing TDP-43 aggregation. Additionally, the role of the 14-amino acid linker in modulating the overall dynamics of TDP-43 suggests that this region could be another promising target for therapeutic intervention. Compounds that stabilize the linker in a specific conformation could alter the aggregation propensity of TDP-43, providing a new approach to disease treatment. The open and flexible conformation of the RNA-free TDP-43 RRMs confirms that the protein is highly dynamic and capable of adopting multiple conformations depending on its interaction partners. This flexibility may be a key factor in its aggregation behavior and its ability to form different types of aggregates under different pathological conditions.

Additionally, we highlight the importance of maintaining EGCG in its reduced and stable form during experiments, as its degradation can confound results. Adding any component without a clear understanding of its structure and stability can completely undermine the rigor of the investigation. Therefore, ensuring that EGCG remains in its stable and reduced form allowed us to draw reliable conclusions about its effects on TDP-43 aggregation. These insights extend our understanding of the mechanism of action of EGCG and its therapeutic potential in TDP-43 proteinopathies.

Future research should focus on bridging the gap between *in vitro* findings and clinical applications to better understand the therapeutic potential of EGCG. The development of novel EGCG derivatives with enhanced binding affinity and specificity for TDP-43 RRMs could also be a promising avenue for therapeutic development. Finally, understanding the interplay between TDP-43 aggregation, RNA binding, and cellular stress responses will be crucial for developing comprehensive therapeutic strategies for ALS and related disorders.

## Methods

### Production of Recombinant TDP-43 RRMs

In the present study we used a recombinant TDP-43 deletion mutant comprising residues K102-Q269, which includes both RRM1 and RRM2 (TDP-43 RRMs). The recombinant protein was expressed and purified as previously described with minor adjuments.^27^ Briefly, TDP-43 RRMs was expressed in *E. coli* Rosetta2(DE3) cells as a fusion protein with a TEV cleavable N-terminal SUMO solubilization domain carrying a 6xHis tag. Protein expression was induced by adding 0.5 mM Isopropil-β-D-1-tiogalattopiranoside, and carried out overnight at 18 °C. Cells were then collected by centrifugation and resuspended in lysis buffer (10 mM potassium phosphate buffer pH 7.2, 150 mM KCl, 5% v/v glycerol, 1 mg/ml lysozyme, Roche complete™ EDTA-free Protease Inhibitor, 5 mM MgCl_2_, 1 µg/ml DNase I, and 1 µg/ml RNaseA). Cells disruption was performed by 1 h incubation at RT followed by probe-sonication. Soluble 6xHis-SUMO tagged TDP-43 RRMs was recovered in the supernatant of the lysate by centrifugation at 70,000 rcf for 45 min at 4 °C. Isolation of the target protein was performed by immobilized metal ion affinity chromatography loading the clarified lysate onto a Cytiva 5 ml pre-packed Ni Sepharose affinity column for capturing. Protein elution was performed by a 0–500 mM imidazole linear gradient. The 6xHis-SUMO protein was then cleaved by incubation with TEV protease (1:2 TEV: Protein molar ratio) for 2 h at RT, followed by overnight at 4 °C, while dialyzing the mixture against 10 mM potassium phosphate buffer pH 7.2, 150 mM KCl. The cleaved protein was recovered into the flow-through of a second nickel affinity chromatography column, and loaded onto a Cytiva HiTrap Heparin column to remove unwanted nucleic acids. The protein was eluted with potassium phosphate buffer with 1.5 M KCl, and submitted to size-exclusion chromatography using a HiLoad 16/60 Superdex 75 prep grade pre-equilibrated in 10 mM potassium phosphate buffer pH 7.2, 150 mM KCl. Protein identity and purity were checked by SDS-PAGE analysis.

For ^15^N-labelled TDP-43 RRMs samples, cell growth and protein expression were conducted in minimal media supplemented with ^15^N-NH_4_Cl as sole source of nitrogen.

### Thioflavin T aggregation assay

TDP-43 RRMs aggregation kinetics was monitored by Thioflavin T (ThT, Sigma-Aldrich T3516) fluorescence emission.^65–67^ ThT fluorescence intensity measurements were performed at 37 °C on protein samples concentrated 15 µM, in 20 mM Hepes buffer pH 7.5 supplemented with 150 mM NaCl, 1mM Dithiothreitol (DTT) and 26 µM ThT. All samples underwent Size Exclusion Chromatography (SEC) immediately before the ThT aggregation assay, to avoid the impact of possible seeds of aggregation and to ensure sample quality. Measurements were performed at least in triplicate under constant agitation at 500 rpm using a CLARIOstar Plus MicroPlate reader (BMG LabTech), incubating the samples in optically clear-bottomed 96-well black plates (Perkin Elmer, 6005225), sealed with a clear adhesive to prevent samples evaporation (Perkin Elmer, 6050185). ThT emission was measured up to 90 h every 30 min at 480 nm under excitation at 440 nm, from the plate bottom. EGCG was added to the protein samples from stock solution at concentrations ranging from 0 to 30 µM. The stock solution was prepared by dissolving EGCG as powder (Sigma-Aldrich, E4143) in 20 mM Hepes buffer pH 7.5, 150 mM NaCl, 1mM DTT, and filtering the solution with 0.22 μm cellulose acetate syringe filters.

### Microscopy images acquisition

Endpoints from ThT aggregation assay were transferred to black, optically clear flat-bottom, 96-well PhenoPlate plates (PerkinElmer, 6055700). Images, both in brightfield and fluorescence mode, were acquired using an Operetta CLS High content screening microscope with a 40x objective. A minimum of 25 fields were acquired *per* well.

### Ligand-based NMR measurements

Saturation Transfer Difference (STD) and Transferred Nuclear Overhauser Enhancement (trNOE) NMR experiments were acquired at 298 K on a Bruker AVANCE 800 MHz NMR spectrometer equipped with cryoprobe.

In the STD-NMR analysis, a 1D ^1^H NMR reference spectrum was first recorded on a sample containing unlabeled TDP-43 RRMs with a 100-fold excess of EGCG. A second 1D ^1^H NMR spectrum was acquired selectively saturating the protein signals for 3 s, on resonance at 0.55 ppm and off resonance at − 40 ppm (saturated spectrum). A spin-lock filter of 40ms was applied to eliminate the protein signals. Subtracting the saturated spectrum from the reference spectrum produced the STD-NMR spectrum.

The NMR experiments 2D NOESY on free EGCG, and trNOE on EGCG in the presence of TDP-43 RRMs were acquired by using standard pulse sequence from Bruker. Mixing time was set at 600 ms and 100 ms for free EGCG and EGCG/protein, respectively.

All spectra were recorded in 10 mM potassium phosphate buffer pH 7.2 and 150 mM KCl supplemented with 0.5 mM of tris(2-carboxyethyl)phosphine (TCEP), and analyzed by Bruker Topspin software.

### Generation of RNA-free TDP-43 RRMs structural model

The RoseTTAFold suite was exploited to quickly and accurately predict the three-dimesional structure of RNA-free TDP-43 RRMs.^54^ Through a “three-track” neural network, this web-based tool is able to process sequence, distance, and coordinate information simultaneously, resulting in the generation of accurate structural models. In the case of RNA-free TDP-43 RRMs, the tool generated five best-scored models with a high confidence value of 0.81.

### NMR backbone assignment

To perform the NMR resonance assignment of the backbone atoms of RNA-free TDP-43 RRMs, 2D ^15^N-HSQC, 3D ^15^N-NOESY-HSQC, and 3D ^15^N-TOCSY-HSQC NMR experiments were acquired at 298 K using a Bruker AVANCE 800 MHz NMR spectrometer equipped with a cryoprobe. All spectra were recorded on a sample of 0.2 mM ^15^N-labelled TDP-43 RRMs in 10 mM potassium phosphate buffer pH 7.2, 150 mM KCl, and 0.5 mM TCEP. Data were processed using NMRpipe^68^ and analyzed by CcpNmr software.^69^

The assignment at pH 6.5 already available on Biological Magnetic Resonance Bank (BMRB, ID 27613),^70^ was used as starting point. About 70% of the amide resonances were transferred by visual inspection. The remaining resonances were assigned by analysis of the 3D experiments.

### NMR thermal coefficient measurements

Amide chemical shift temperature coefficients were calculated for 0.2 mM of ^15^N-labelled TDP-43 RRMs in 10 mM potassium phosphate buffer pH 7.2, 150 mM KCl, 0.5mM TCEP, and 10% D_2_O. A series of ^1^H–^15^N HSQC spectra were recorded at 283, 288, 293, 298, and 303 K using a Bruker AVANCE 800 MHz NMR spectrometer equipped with a cryoprobe. All spectra were referenced to the water signal for each temperature, processed using Bruker Topspin suite, and analyzed by CcpNmr software.^69^

The chemical shift values δ_HN_ of all residues were plotted as a function of the temperature. The data were analyzed assuming that residues with dδ_HN_/dT < − 5 ppb/K form weaker H-bonds and should be considered secondary structure breakpoints or, if in an unstructured region, as promoters of H-bonds with water. Values of dδ_HN_/dT > − 5 ppb/K correspond to the formation of tighter bonds likely involved in secondary structure elements.

### NMR chemical shift perturbation experiments

To identify the EGCG binding surface on TDP-43 RRMs, a series of 2D ^1^H–^15^N HSQC spectra were recorded on 0.2 mM ^15^N-labelled TDP-43 RRMs titrated with increasing concentration of EGCG (used ligand:protein molar ratios 0.0, 0.15, 0.25, 0.35, 0.5, 0.75, 1.0, 1.25, 1.5, 2.0). Chemical Shift Perturbation (CSP) was followed and calculated using the interactive CSP analysis module of CcpNmr software.^69^ Peaks in overlap were excluded from the analysis. The threshold value was set equal to 2σ, indicated as a standard practice.^71^

### Relaxation heteroNOE NMR experiments

Relaxation hetNOE NMR experiments were acquired on 0.2 mM TDP-43 RRMs in 10 mM potassium phosphate buffer pH 7.2, 150 mM KCl, 0.5mM TCEP, and 10% D_2_O, both in the absence of EGCG and in the presence of 2-fold excess of EGCG. For each sample, two bi-dimensional ^15^N spectra were recorded: a reference spectrum and a spectrum recorded with an initial proton saturation of 8s. The NOE values were then determined from the ratios of the average intensities of the peaks with and without proton saturation. The data were analyzed by the Dynamic Center tool provided by Bruker topspin platform.

### Molecular docking

The TDP-43 RRMs model 1 predicted by RoseTTAFold was arbitrarily selected to perform experimentally-driven 3-body-docking calculation with two molecules of EGCG (PubChem ID 65064). The docking was performed on the integrative modelling platform HADDOCK2.4.2.^60,61^ The simulation was launched by setting as active those residues with a CSP value higher than 2σ (*vide infra*) and forming a contiguous surface on each RRM domain. More specifically, residues L109, G110, L111, F147 and D174 laying on RRM1 were specified to interact with one molecule of EGCG, while V193, F194, V220, F231, A260 laying on RRM2 were specified to interact with a second molecule of EGCG. Two sets of experimental AIR restraints were introduced in TBL format, generated using HADDOCK server tool http://alcazar.science.uu.nl/services/GenTBL/. The restraints were implemented at different stages of the calculations. During the rigid-body docking, the two CSP-guided binding regions on the protein and both the ligand molecules were selected as active. It followed the flexible refinement stage, where the two CSP-guided binding regions on the protein were turned inactive. After a third stage of water refinement, the RMSD was implemented as clustering method with a cut-off of 2.0 Å. For each pose, residues located at a maximum distance of 3 Å from EGCG structure were analysed using PyMOL suite.^72^

### Molecular dynamic simulation

Classical molecular dynamics (MD) simulations were performed with the GROMACS 2022 software package (10.5281/zenodo.6103835), using CHARMM36 all-atom force field to generate the protein topology.^73^ The ligand topology was obtained through a web tool based on the same force field https://cgenff.com/. The best structure of selected HADDOCK clusters 1 and 2 were used to build the systems. Structures were solvated in a cubic box with explicit water molecules (TIP3P). Counterions were added to neutralize the charge of the system. The systems were subjected to two equilibration steps after a short minimization using the steepest descent algorithm with a force convergence criterion set to 1000 kJ/mol·nm^2^. The first step was carried out to equilibrate and it was performed by applying positional restraints on the protein and the ligands. The second step was carried out to equilibrate the pressure and it was performed by coupling the systems with the Parrinello-Rahman barostat.^74,75^ The production run was performed at constant pressure (1 atm) and temperature (300 K) for 100 ns using periodic boundary conditions and the velocity-rescaling thermostat.^76^ LINCS algorithm^77^ was used to constrain the bonds involving hydrogen atoms, while the particle mesh Ewald method^78^ was used to account for long-range electrostatic interactions with a cutoff of 10 Å. An integration time step of 2 fs was used in all simulations. All the trajectories were inspected and analysed with Gromacs utilities. Root-mean-square deviation (RMSD) and root-mean-square fluctuations (RMSF) were measured with tools implemented in GROMACS 2022. Hydrogen bond occupancy and distances were measured using VMD.^79^

## Electronic supplementary material

Below is the link to the electronic supplementary material.

Supplementary Material 1

## Data Availability

All data are available from the corresponding author on reasonable request.
