# Supplementary material for: Epigallocatechin-3-gallate binds tandem RNA recognition motifs of TDP-43 and inhibits its aggregation
Source: Sci Rep. 2025 May 23;15:17879. doi: 10.1038/s41598-025-02035-6 (PMC12098689; doi:10.1038/s41598-025-02035-6)
Supplement: Supplementary file 1 — Supplementary Material 1 [file 41598_2025_2035_MOESM1_ESM.docx]

**SUPPLEMENTARY INFORMATION**

**Epigallocatechin-3-gallate binds tandem RNA Recognition Motifs of TDP-43 and inhibits its aggregation**

Maria Agnese Morando^1,§^, Vito D’Alessandro^1,2,§^, Angelo Spinello^3^, Martina Sollazzo^1,3^, Elisa Monaca^1^, Raffaele Sabbatella^1^, Maria Concetta Volpe^4^, Francesca Gervaso^5^, Alessandro Polini^5^, Sarah Mizielinska^6,7^, Caterina Alfano^1*^

^1^Structural Biology and Biophysics Unit, Fondazione Ri.MED, Palermo 90133, Italy

^2^Department of Mathematics and Physics "E. De Giorgi", University of Salento, Lecce 73100, Italy

^3^Department of Biological, Chemical and Pharmaceutical Sciences, University of Palermo, Palermo 90100, Italy

^4^Fondazione Ri.MED, Palermo 90133, Italy

^5^CNR Nanotec - Institute of Nanotechnology, Campus Ecotekne, Lecce 73100, Italy

^6^UK Dementia Research Institute at King's College London, London, UK.

^7^Department of Basic and Clinical Neuroscience, Institute of Psychiatry, Psychology and Neuroscience, King's College London, London, UK

^§^These authors equally contributed to the work

*To whom correspondence should be addressed

[calfano@fondazionerimed.com](mailto:calfano@fondazionerimed.com)

**Keywords**: EGCG stability, TDP-43, ALS, protein aggregation, RNA-binding proteins, protein-ligand interaction

**
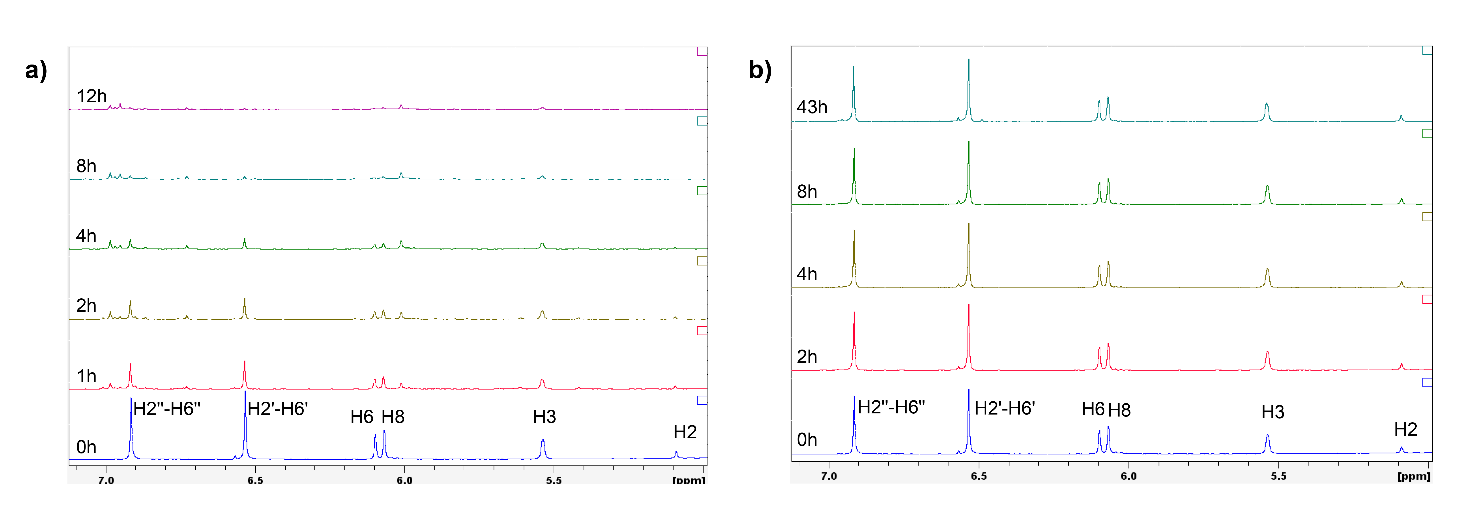
Suppl. Fig. 1: Assessment of EGCG stability by 1D ^1^H NMR analysis.** 1D ^1^H NMR spectra of 0.2 mM EGCG in 10 mM potassium phosphate pH 7.2 and 150 mM KCl, were registered at several time points to investigate the stability of the compound over time. A) Spectra acquired in the absence of reducing agents. NMR signals of EGCG drastically reduce over time and completely disappear after 8 hours, indicating very low stability of the compound probably due to oxidation events.^1,2^ As expected, the solution within the NMR tube changed color during the observation period, from transparent to brownish. B) Spectra acquired in the presence of TCEP as a reducing agent to prevent EGCG oxidation. No change in the NMR spectra was observed up to 43 hours, demonstrating TCEP capability to preserve EGCG stability over time.


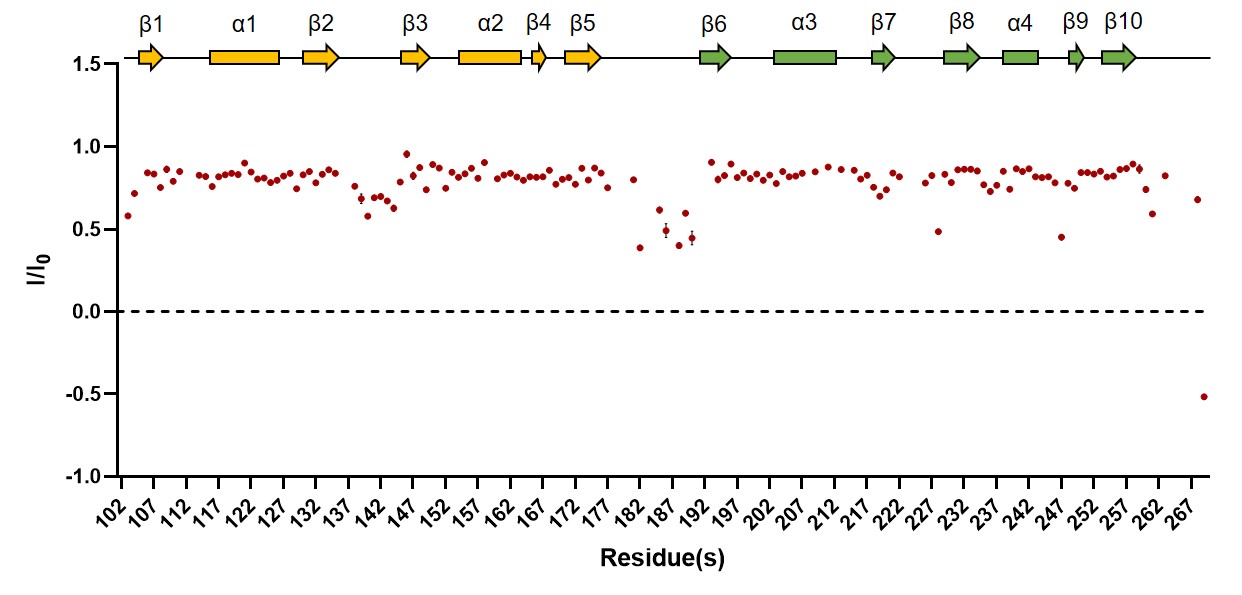


**Suppl. Fig. 2:** **Dynamic features of RNA-free TDP-43 RRMs**. ^1^H-^15^N hetNOE measured at 298 K for ^15^N-labeled TDP-43 RRMs at 18.8 T. X-axis represents the residue number. Y-axis represents the ^15^N hetNOE values (I/I_0_). Each data point corresponds to a specific residue in the protein sequence. Higher NOE values (close to 1) indicate less mobility and are displayed by those residues involved in structured regions like alpha-helices and beta-sheets. Lower NOE values indicate higher mobility and are displayed by the residues within the loop between RRM1 and RRM2 and the C-terminal tail.

**
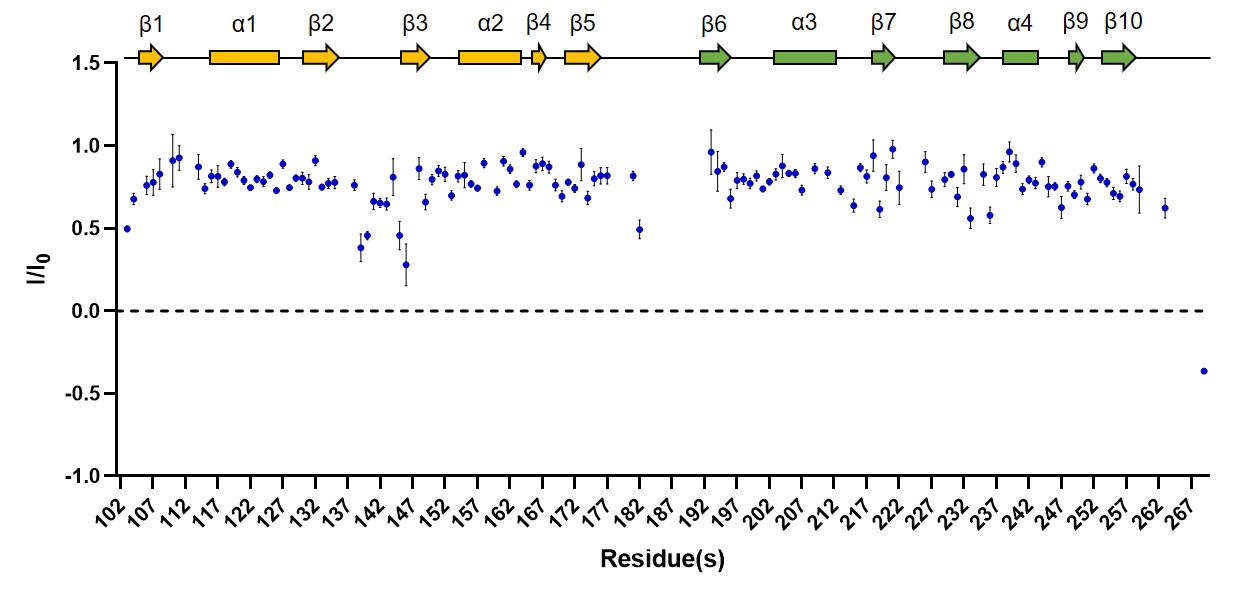
**

**Suppl. Fig. 3:  Dynamic features of EGCG-bound TDP-43 RRMs.** ^1^H-^15^N hetNOE measured at 298 K for ^15^N-labeled TDP-43 RRMs in the presence of 2-fold EGCG excess. X-axis represents the residue number. Y-axis represents the ^15^N hetNOE values (I/I_0_). Each data point corresponds to a specific residue in the protein sequence. The signals of most of the residues lying within the flexible linker disappeared, suggesting that they experience greater motion with respect to the ECGC-free state of the protein and are not then involved in the binding with the ligand. Indeed, a direct binding to EGCG would have provided these residues with a greater rigidity, making their signals visible.


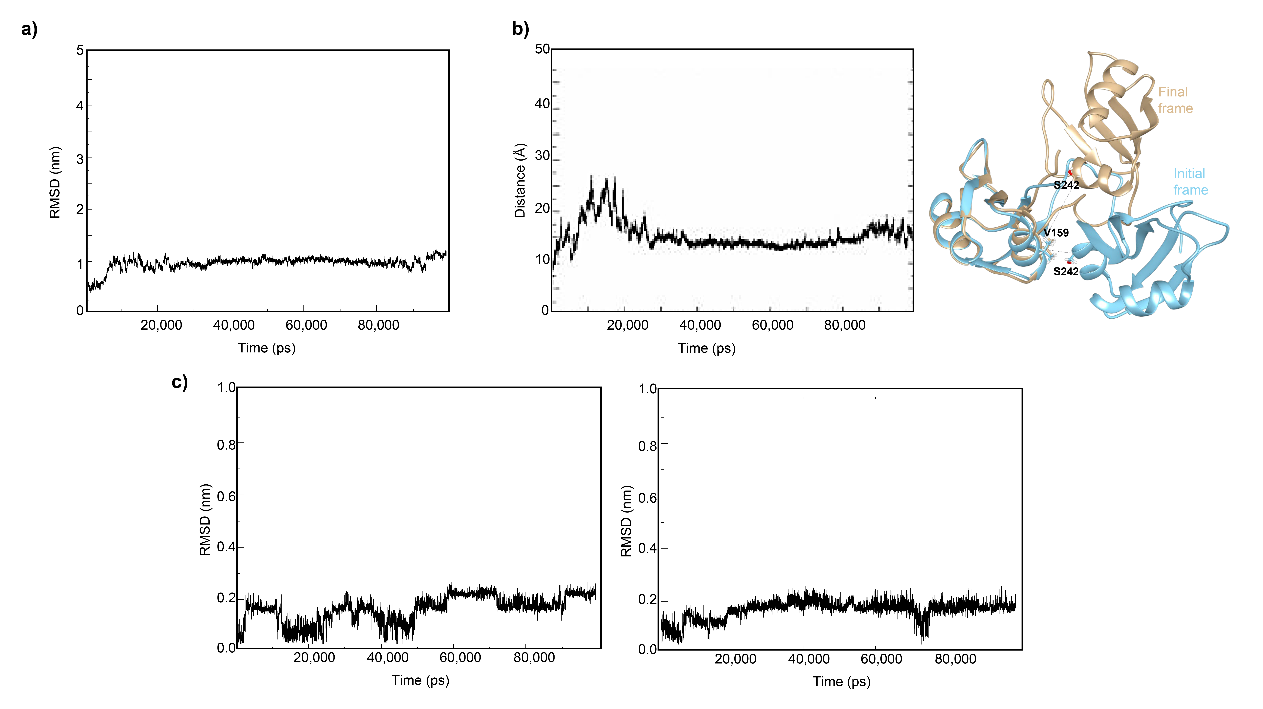


**Suppl. Fig. 4: Parameters from Molecular Dynamic simulations on HADDOCK Cluster 3.** a) RMSD for the protein structure over 100ns of simulation from the starting HADDOCK model. b) Distance between Cα of V159 and Cα of S242 measured along the simulation. The two RRM domains assume different orientations with respect to the initial frame, moving away from each other. This distance correlated with the RMSD fluctuations observed in a). c) RMSD along the 100ns simulation of EGCG molecule bound to RRM1 (left) and RRM2 (right).

**REFERENCES**

1. Mizooku, Y., Yoshikawa, M., Tsuneyoshi, T. & Arakawa, R. Analysis of oxidized epigallocatechin gallate by liquid chromatography/mass spectrometry. *Rapid Commun Mass Spectrom* **17**, 1915–1918 (2003).

2. Ouyang, J., Zhu, K., Liu, Z. & Huang, J. Prooxidant Effects of Epigallocatechin-3-Gallate in Health Benefits and Potential Adverse Effect. *Oxid Med Cell Longev* **2020**, 9723686 (2020).
